# Chemical–Physical Properties of Red Palm Oils and Their Application in the Manufacture of Aerated Emulsions with Improved Whipping Capabilities

**DOI:** 10.3390/foods12213933

**Published:** 2023-10-27

**Authors:** Ziwei Gao, Yun Zhu, Jun Jin, Qingzhe Jin, Xingguo Wang

**Affiliations:** State Key Laboratory of Food Science and Resources, School of Food Science and Technology, Jiangnan University, Wuxi 214122, China; 6210112014@stu.jiangnan.edu.cn (Z.G.); 7220111015@stu.jiangnan.edu.cn (Y.Z.); jqzwx12@163.com (Q.J.); wangxg1002@gmail.com (X.W.)

**Keywords:** red palm oil, oxidative stability, mango kernel fat, aerated emulsion, foam stability, whipping capability

## Abstract

Red palm oil (RPO), which is rich in micronutrients, especially carotenoids, is different from its deodorized counterpart, palm oil. It is considered as one of the most promising food ingredients, owing to its unique compositions and nutritional values, while its usage could be further developed by improving its thermal behaviors. In this article, two typical commercial RPOs, HRPO (H. red palm oil) and NRPO (N. red palm oil), were evaluated by analyzing their fatty acids, triacylglycerols, micronutrients, oxidative stability index (OSI), and solid fat contents (SFCs). Micronutrients, mainly carotenes, tocopherols, polyphenols, and squalene, significantly increased the oxidative stability indices (OSIs) of the RPOs (from 10.02 to 12.06 h), while the OSIs of their micronutrient-free counterparts were only 1.12 to 1.82 h. HRPO exhibited a lower SFC than those of NRPO. RPOs softened at around 10 °C and completely melted near 20 °C. Although the softening problem may limit the usages of RPOs, that problem could be solved by incorporating RPOs with mango kernel fat (MKF). The binary blends containing 40% RPOs and 60% MKF exhibited desirable compatibilities, making that blend suitable for the manufacture of aerated emulsions with improved whipping performance and foam stabilities. The results provide a new application of RPOs and MKF in the manufacture of aerated emulsions with improved nutritional values and desired whipping capabilities.

## 1. Introduction

Red palm oil (RPO) is a natural oil obtained from the fruit of oil palm (*Elaeis guineensis*). It is produced by physical refining, retaining its natural fat-soluble tocopherols, tocotrienols, and carotenoids. In general, the special red oil contains highly bioavailable β-carotene and vitamin A, and is used as an antiatherogenic, an antihypertensive, an anticarcinogen, and an antidiabetic, and to prevent vitamin A deficiency [1,2]. In contrast, in chemical refining, high-temperature steam deodorization destroys carotene [3]. The fractionation of triacylglycerols is the most prevalent modification approach for enhancing palm oil utilization, increasing the usefulness of oils and fats, and allowing them to be used in more edible applications [4,5]. Therefore, RPO is generally fractioned as olein and stearin, which are consumed around the world in baking, snacks, frozen foods, and chocolates, due to their natural taste, texture, and modified melting properties [4,5].

RPO has a balanced fatty-acid composition of about 50% saturated fatty acids, 40% monounsaturated fatty acids, and 10% polyunsaturated fatty acids, making it possible for the formulation of tailored shortenings, confectionary fats, and frying oils in the food industry [6]. In particular, RPO provides additional nutritional values, based on its high levels of micronutrients. However, the usages of RPO as a specialty fat are generally limited in tropical and subtropical areas with hot weather or significant temperature fluctuations, because of their softening properties at around 32–35 °C, which result in quality defects of related products, especially poor whipping capabilities of aerated emulsions such as whipping creams. Aerated emulsions are multiphase systems, in which an oil phase is dispersed into a continuous phase. In such systems, the presence of fat crystals in the dispersed phase can cause specific instabilities that are known as partial coalescence [7]. They can be transformed into foam structures by whipping, and bubbles are incorporated and surrounded by partially coalesced fat globules after whipping [8]. The foam structures are currently widely used in various foods such as cakes, desserts, pastries, and toppings [9]. It is necessary to improve the heat-stable properties of RPO by incorporating it with hard fats to make it suitable for producing high-quality food products for consumption in tropical and subtropical areas.

In the present study, two typical commercial RPOs were analyzed according to their triacylglycerol and micronutrient compositions, as well as their nutritional values, and their heat-stable properties were modified by incorporating them with a potential tropical fat, i.e., mango kernel fat (MKF) and by evaluating their solid-fat contents (SFCs) and compatibilities. Potential usages of the binary blends in manufacturing aerated emulsions were further analyzed, based on their melting behaviors, whipping capabilities, and foam structures. The aim of this research was to examine the possibility of improving the whipping capabilities of aerated emulsions formulated from binary mixtures of RPOs and MKF. The results improve the whipping performance and the nutritional values of vegetable fat-based aerated emulsions by regulating the amounts of RPOs and MKF.

## 2. Materials and Methods

### 2.1. Materials

H. red palm oil (HRPO) and N. red palm oil (NRPO) were produced from palm-oil-processing factories in Malaysia. Anhydrous milk fat (AMF) was obtained from the Mengniu Dairy Products Co., Ltd. (Inner Mongolia, China). MKF was refined from mango kernels, according to our previous report [10]. Mango kernels were donated by a local mango-juice-processing company in Guangxi, China. Standards of 37 fatty acid methyl esters, α-, β-, γ-, δ-tocopherols (purity > 95%), and 5α-cholestane were purchased from Sigma-Aldrich Chemical Co. Ltd. (Shanghai, China). Other reagents and solvents were provided by Sinopharm Chemical Regent (Shanghai, China).

### 2.2. Determination of Fatty Acid Composition

Fatty acid methyl esters of HRPO and NRPO were prepared, as per our previous report, with modification [11], and evaluated by a gas chromatograph system (7820A, Agilent, Santa Clara, CA, USA) equipped with a hydrogen flame ionization detector and a DB-Fast FAME capillary column (30 m × 250 μm, 0.25 μm, Agilent, CA, USA). The conditions of analysis were as follows: carrier gas (nitrogen), 1 mL/min; injector temperature, 250 °C; detector temperature, 260 °C; split ratio, 1:100; injection volume, 1.0 μL. The initial column temperature was 80 °C (0.5 min); it was programmed up to 165 °C at a rate of 40 °C/min and maintained for 1 min, then programmed up to 220 °C at a rate of 2 °C/min and maintained for 2 min. The running time of the program was about 32 min. The fatty acid compositions were identified by comparing the retention times of the standards and their percentages were reported in terms of the relative proportions.

### 2.3. Determination of Triacylglycerol Composition

Triacylglycerol species and compositions of HRPO and NRPO were determined by a gas chromatograph (7890A, Agilent, USA) equipped with a DB-17HT capillary column (0.15 μm, 0.25 mm × 15 m, Agilent, USA) and a hydrogen flame ionization detector (FID). The operating conditions for analysis were as follows: nitrogen (carrier gas), 1.0 mL/min; split ratio, 1:100; injector temperature, 360 °C; detector temperature, 375 °C. The initial column temperature was 250 °C; it was programmed up to 340 °C at a rate of 5 °C/min and then maintained at 340 °C for 45 min. The sample concentration in hexane was 25 mg/mL and the injection volume was 1.0 μL. Triacylglycerols were identified by carbon numbers and by the unsaturation degree, as well as by comparing the retention time of the triacylglycerol standards. The content of each triacylglycerol was reported in terms of the relative proportion.

### 2.4. Determination of Sterol and Squalene

The sterol and squalene of HRPO and NRPO were analyzed by a gas chromatograph–mass spectrum system (Thermo Fisher, Waltham, MA, USA) equipped with an FID. First, 200 mg of each fat sample was mixed with 0.5 mL of 0.1 mg/mL 5α-cholestane and 3 mL of 2 mol/L KOH-CH_3_CH_2_OH. The mixture was heated at 85 °C for 1 h and then cooled, and 2 mL of distilled water and 5 mL of hexane were added three times to extract the supernatant liquid. The product was then dried by nitrogen and silylated with 200 μL Bis (trimethysiyl) trifluoroacetamide (BSTFA) + trimethylsilyl (TMCS) at 75 °C for 30 min, and finally dissolved by 1 mL hexane. The conditions for analysis were as follows: DB-5 capillary (0.25 μm, 30 m × 0.25 mm, Agilent, USA). The initial column temperature was set at 200 °C for 0.5 min, then increased to 300 °C at a rate of 10 °C/min and maintained for 18 min. Both the detector temperature and the injector temperature were 280 °C. The nitrogen (carrier gas) was 1.2 mL/min and the split ratio was 1:100. The ion source and transmission line temperatures were 250 °C. The ionization mode was electron ionization (EI) and the mass range was 50–550 *m*/*z*. The contents of sterol and squalene were reported in mg/kg.

### 2.5. Determination of Tocopherols

Tocopherols of HRPO and NRPO were determined by a high-performance liquid chromatographic system (LC-20 AT, Shimadzu, Kyoto, Japan) equipped with an ultraviolet detector (SPD-20A, Shimadzu, Japan) set at 295 nm. About 50 mg of the fat sample was diluted into 10 mL of hexane. Then, 20 μL of the solution was injected into the machine, and the separation was performed on a silica column (5 μm, 4.6 × 250 mm, Hanbon, Huaian, China) using hexane/isopropanol (98.5/1.5, *v*/*v*) as the mobile phase, with a rate of 1.0 mL/min. The column temperature was 30 °C and the determination wavelength was 295 nm. Tocopherols, including α-, β-, γ-, and δ-isomers, were identified and quantified by comparing the standards, and their contents were reported in mg/kg.

### 2.6. Determination of Carotene

Carotene contents of HRPO and NRPO were measured, according to the standard method, with some modifications [12]. About 5 g of the samples were saponified with 5 mL 50% ethanolic KOH by heating at 50 °C in the dark in a water bath under a stream of nitrogen for 45 min. The saponified sample was then cooled to room temperature and extracted with 50 mL portions of petroleum ether until the supernatant became colorless. The pooled petroleum ether extracts were washed four times with 50 mL portions of distilled water and dried over anhydrous sodium sulfate. The extract was then dried in a rotary evaporator at 50 °C. The residue was dissolved in a known volume of mobile phase for HPLC analysis.

### 2.7. Determination of Total Polyphenols

The total polyphenol contents of HRPO and NRPO were measured by the Folin–Ciocalteu method described previously, with some modifications [13]. First, 1 g of the fat sample was extracted with 5.0 mL of methanol three times, and 1 mL combined supernatants was mixed with 1 mL of Folin–Ciocalteu reagent and 3 mL of sodium carbonate solution (10% *v*/*v*). The mixture and the distilled water were then added into a 10 mL volumetric flask. After 2 h of incubation in the dark, the absorbance at 760 nm was measured by a microplate reader (Spectra Max M2, Molecular Devices, Sunnyvale, CA, USA). The total polyphenol contents were expressed as a gallic acid equivalent (mg GAE/kg oil).

### 2.8. Determination of Oxidative Stability Indices

The oxidative stability indices (OSIs) of the HRPO and the NRPO were measured by a Metrohm Rancimat model 743 (Herisau, Switzerland), according to the AOCS Cd 12b-92 method [14]. About 5 g of oil was heated at 120 °C, and 20 L/h of the cleaned and dried air was bubbled into the hot sample. Effluent air containing volatile organic acids from the sample was collected in a measuring vessel containing 50 mL of distilled water. The conductivity of the water was measured automatically as oxidation proceeded, and the result was recorded in hours (h). The removal of micronutrients in RPOs was carried out according to the method reported by Tang et al. [15]. Minor constituents in the RPOs were entirely removed by passing the oil through a chromatographic glass column (30 cm × 50 cm) packed with alumina, silicic acid, diatomite, activated carbon, and silica gel (5:2:1:2:5) homogenized with 100 mL n-hexane. The bottom layer was padded with a layer of absorbent cotton in order to prevent leakage of the packing. The height of the packing in the middle layer of the chromatographic column was 30 cm. The residual n-hexane was removed by rotary steaming and nitrogen blowing, and the whole process was hidden from light. The determination method of the OSIs of the RPOs without micronutrients was the same as above.

### 2.9. Evaluation of Solid-Fat Content and Compatibility

The SFCs of the HRPO and the NRPO were determined according to the AOCS Official Method Cd 16b-93 [16]. A fat sample (3–5 mg) was poured into each nuclear magnetic resonance (NMR) tube and melted at 80 °C to erase crystal memory. The SFC of the fat was determined at temperatures ranging from 0 to 40 °C at 5 °C intervals by equilibrating the NMR tubes at these temperatures for 30 min before measurement [17].

The HRPO and the NRPO were then blended with MKF in increments of 20%. Specifically, HRPO-MKF I, HRPO-MKF II, HRPO-MKF III, and HRPO-MKF IV were prepared by blending HRPO with MKF ranging from 20:80 to 80:20 (with 20% intervals). Similarly, NRPO-MKF I, NRPO-MKF II, NRPO-MKF III, and NRPO-MKF IV were binary blends containing NRPO and MKF ranging from 20:80 to 80:20 (with 20% intervals). The SFC of the binary blends was measured as described above. The compatibilities of the binary blends consisting of the RPOs and MKF were analyzed by introducing their iso-solid phase diagrams [18].

ΔSFC represents the compatibilities of the binary blends, which were calculated from the difference between the measured SFC (SFCm) and the theoretical (SFCt), as in the following equation:

ΔSFC = SFCm − SFCt

where SFCt = x% SFCx + y% SFCy; x% and y% are the mass fractions of the two components in the binary system, respectively; and SFCx and SFCy are the measured SFC values of the two components at the measured temperature. Theoretically, the closer ΔSFC is to 0, the better the compatibility.

### 2.10. Determination of Fractal Dimensions

The fat crystal microstructure of each fat sample was observed by a polarized light microscope equipped with a spot idea camera (PL-180; Shangguang, Shanghai, China). One droplet of the melted sample was taken by a capillary and placed on a preheated glass slide, then covered with a coverslip. The samples were stored at 4 °C for 24 h to finish the crystallization before observation and image collection. To obtain the fractal dimension (D*f*), the images were processed by imageJ 1.36b software (National Institutes of Health, Bethesda, MD, USA), according to the method reported by Liu et al. [19]. Each experiment was repeated three times.

### 2.11. Evaluation of Whipping Capabilities of Aerated Emulsions

#### 2.11.1. Whipping Time

The aerated emulsions were whipped using a household kitchen mixer (DDQB01K1, Bear, Foshan, China) at a speed of 800 rpm, and the whipping time was recorded when foam formed a soft spike with maximum firmness.

#### 2.11.2. Measurement of Overrun and Serum Loss

Overrun indicates the ability to introduce air into the aerated emulsions. The overrun was determined using the method described by Scurlock [20]. The overrun was then calculated by the following equation:

$$\mathrm{Overrun}\;(\%)=\frac{M_{1}-M_{2}}{M_{2}}\times 100$$

where *M*_1_ and *M*_2_ represent the mass of the unwhipped sample (g) and the whipped sample (g), respectively.

Whipped cream (around 5 g) was transferred to a 50-mesh sieve at an ambient temperature for 2 h. The serum loss was calculated by the equation below:

$$\mathrm{Serum}\;\mathrm{loss}\;(\%)=\frac{m_{1}}{m_{2}}\times 100$$

where $m_{1}$ and $m_{2}$ are the mass of serum loss (g) and the mass of whipped creams (g), respectively.

#### 2.11.3. Cream Performance after Storage

The freshly whipped sample was stacked into a forming topping, using a nozzle, and set on a petri dish placed at 20 °C for 0 h and 2 h, respectively. The degree of roughness of the cream surface was observed, and a small photo studio was employed to photograph their appearance.

### 2.12. Texture Analysis

A TA-XT Plus (Stable Microsystems, Surrey, UK) equipped with an aluminum cylinder probe (model P-25; diameter: 25 mm) was employed to analyze the textural properties of the whipped cream. The properties were measured by the method reported by Zhai et al. [21], with slight modifications. The sample (150 mL) was placed in a cylindrical container and the surface was scraped flat with a spatula. The probe penetrated into the sample to a depth of 25 mm at a rate of 1 mm/s, and the 5 g trigger force exerted on the probe was automatically recorded. Three parameters—firmness, consistency, and cohesiveness—were identified immediately after removal from the fridge (4 °C).

### 2.13. Observation of Microstructure of the Whipped Cream

Polarized light microscopy was applied to visualize air bubbles. The samples were prepared according to the method reported by Xie et al. [22]. The foam structure was observed using polarized light and a bright field.

### 2.14. Statistical Analysis

All the experiments were carried out in triplicates, and the results were reported as mean ± standard deviation. The data were subjected to statistical analysis, using SPSS 27.0 (IBM, New York, NY, USA) to analyze variance (ANOVA). The significance of differences among the mean values was identified at a level of *p* < 0.05.

## 3. Results and Discussion

### 3.1. Fatty Acids and Triacylglycerols of Red Palm Oils

The fatty acid compositions of each fat sample are presented in Table 1. The most abundant fatty acids in HRPO and NRPO were palmitic acid (C16:0, 34.95–42.96%), oleic acid (C18:1, 38.99–42.27%), and linoleic acid (C18:2, 10.70–11.57%); in addition, the fat contained appreciable quantities of stearic acid (C18:0, 3.87–5.83%). The total unsaturated fatty acids accounted for 49.69–54.40% of the fat. Such a unique composition pattern was in agreement with that of other reported red palm oils [23]. The dominant fatty acids present in MKF were stearic acid (C18:0, 47.19%) and oleic acid (C18:1, 38.16%). The contents of palmitic acid and steric acid were significantly different in the RPOs and the MKF. Moreover, HRPO-MKF II and NRPO-MKF II were rich in steric acid (C18:0, 28.44–30.31%) and oleic acid (C18:1, 38.58–40.60%), while HRPO-MKF IV and NRPO-MKF IV were dominated by palmitic acid (C16:0, 29.88–35.78%) and oleic acid (C18:1, 38.98–41.76%). The difference in fatty acid compositions influences the fat-crystallization properties, as will be demonstrated in a following study by SFCs and fat-crystal structures.

Table 2 shows the triacylglycerol compositions of the fat samples. POP (22.42–29.31%), POO (18.39–23.10%), and PLP (15.12–15.15%) were the primary triacylglycerols in the RPOs, accounting for over 55% of the total amounts, which had already been confirmed by previous researchers [24]. The presence of 24.95–27.11% of triacylglycerols containing two and three unsaturated fatty acids in RPOs, e.g., POO, SOO, and OOO, may indicate the softer textures of related products. The contents of triacylglycerols containing two and three unsaturated fatty acids in MKF were significantly higher than those of RPOs. Fats enriched with SOS are suggested to improve the thermal properties of fat crystals [25]. In this case, the major TAGs in the MKF were SOS (31.91%), SOO (23.86%), and OOO (9.19%). The SOS and SOO levels in the binary blends decreased from 19.43–19.83% and 15.09–15.12% to 7.48–7.88% and 7.26–7.29%, respectively, with an increase in the ratio of the RPOs.

### 3.2. Micronutrients of Red Palm Oils

RPOs have distinctive flavors and are rich in phytonutrients such as sterol, tocopherol, carotene and polyphenol [26]. The contents of sterol, squalene, tocopherol, total polyphenol, and carotene in HRPO and NRPO are shown in Table 3. 

Sterol is an important constituent of the unsaponifiable of oils and its level in palm oil is about 300–636 mg/kg [27,28]. The HRPO and the NRPO contained 662.96 and 388.14 mg/kg of sterols, respectively. Sitosterol is the most abundant sterol in both RPOs. The content of sitosterol in the HRPO was 440.85 mg/kg, followed by campesterol (142.03 mg/kg) and stigmasterol (74.29 mg/kg). Similar results were demonstrated by Santos et al. [29]. The sterol contents of RPOs depend on the refining temperature; the contents are in the range of 219.9 to 293.6 mg/kg [30]. Because phytosterols are functional ingredients, RPO can be regarded as a desirable edible oil in terms of nutrition. Squalene, a triterpenoid compound with six double bonds, is a biogenic precursor of sterols. It is an effective singlet oxygen quencher [31]. The HRPO and the NRPO contained 28.05 and 52.80 mg/kg of squalene, respectively. In general, squalene is a source of natural nutrients for skin health [32].

The RPOs exhibited desirable antioxidant abilities and radical scavenging activities, with contributions by phenolic compounds [33]. Natural tocopherols, also known as vitamin E, are excellent radical chain-breaking antioxidants. In general, they release active hydrogen of the sixth hydroxyl group on their oxanaphthalene ring, trap free radicals, and form stable compounds with ROO· or R·, thereby blocking free radical chain reactions [34]. The HRPO and the NRPO contained 473.43 and 327.93 mg/kg of tocopherols, respectively, and were dominated by α-tocopherol (137.23–316.70 mg/kg). The data were in accord with a previous report, which found that red palm oil is rich in α-tocopherol (173.04 mg/kg) [35].

Carotenoids, such as α-carotene, β-carotene, and retinyl palmitate, are typical micronutrients that are present in RPOs, contributing to the brilliant red color and to improving the nutritional values and the oxidative stabilities of the oils [36]. Previous research demonstrated that RPO was suitable for use as a frying medium, due to the high heat-stability contributed by carotenoids [37]. Carotenes are types of carotenoids that do not contain oxygen, including α-carotene, β-carotene, γ-carotene, and lycopene. There are multiple double bonds in their structures. As shown in Table 3, the RPOs contained 91.48–154.04 mg/kg of carotenes. The levels are about 15 times more retinol equivalents than those of carrots, 300 times more than those of tomatoes, and 44 times more than those of leafy vegetables [38]. Trace amounts of carotenes could be found in most of the refined palm oils, the carotenes of which are generally removed by deodorization in traditional refining [39]. In contrast, the concentrations in RPOs were significantly higher than those in common vegetable oils (less than 10 mg/kg), such as olive oil and canola oil [40,41].

Polyphenol is a generic term for plant components with multiple hydroxyl phenols. The unique polyphenol structure endows such components with unique functional activities. Polyphenols are complex mixtures of compounds, including oleuropein, 4-hydroxyphenylethanol (tyrosol), 3,4-dihydroxy-phenylethanol (hydroxytyrosol), 4-hydroxyphenylacetic acid, protocatechuic acid, and syringic acid. Phenolic compounds in vegetable oils are free-radical scavengers [42]. RPOs contained 45.00–50.00 mg/kg of total polyphenols, which could make certain contributions to the oxidative stabilities. Their levels were higher than those of soybean oil (3–4 mg/kg), but lower than those of virgin olive oil (200 mg/kg) [43].

### 3.3. Oxidative Stabilities of Red Palm Oils

The OSI is an important parameter for evaluating the shelf life of fats and oils. It is not only related to the saturation degree of fatty acids, but also to micronutrient species and their concentrations [44]. Table 4 shows the OSIs of the two RPOs and their counterparts without micronutrients. OSIs of 10.02–12.06 h were detected in the HRPO and the NRPO, which were significantly higher than those of their micronutrient-free counterparts (1.12–1.82 h). This could be explained by the fact that the presence of micronutrients (especially tocopherols, carotenes, polyphenols, and squalene) played important roles in improving the oxidation stabilities of the RPOs. Previous studies showed that polyphenols scavenge the DPPH and other oxygen-free radicals and that polyphenols are effective stabilizers of α-tocopherol during RPO heating [45,46]. Tocotrienols occupy 70% of tocopherols in palm oil; the unique structure of tocotrienols is a short tail with three double bonds, which allows tocopherols to have high antioxidant potential [47,48]. 

The OSIs of the RPOs were also significantly higher than those of common commercial oils detected at 120 °C, such as 3.05 h for sunflower oil, 5.30 h for corn oil, 3.66 h rice bran oil, 4.10 h for peanut oil, and 3.87 h for canola oil [49,50,51,52,53]. This indicated that the RPOs are suitable for the manufacture of food products with high oxidative stabilities. The Pearson correlation analysis for oxidative stability and micronutrients is shown in Table 5. Squalene exhibited the strongest association among all the micronutrients (*p* < 0.01), followed by the total polyphenol for the RPOs (*p* < 0.05). However, sterol, tocopherol, and carotene showed negative correlations in this case. This could be explained as synergistic and antagonistic effects among these components in the oils.

### 3.4. Solid-Fat Contents of Red Palm Oils

**Figure 1:**
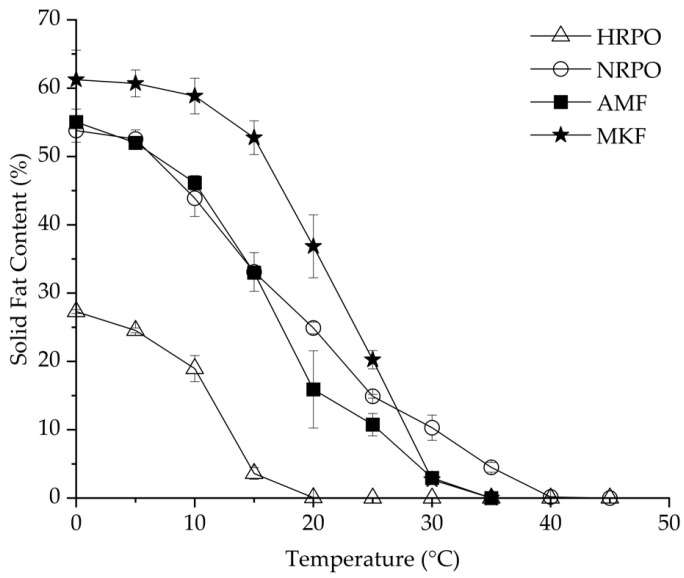
Solid-fat contents of red palm oils, mango kernel fat, and anhydrous milk fat.

RPOs could be used as cooking or frying oils, shortenings, and confectionary fats, based on their SFC profiles [54]. SFC is one of the most important parameters to evaluate the heat-stable properties of fat [55]. Figure 1 shows SFC changes of HRPO, NRPO, MKF, and AMF, ranging from 0 to 40 °C. The HRPO showed the lowest SFC values at all the studied temperatures, decreasing from 25% to zero from 0 to 20 °C. Therefore, it was suggested for use as a cooking oil. In contrast, the NRPO exhibited a relative wide plasticity range, from 0 to 40 °C. The values were close to those of AMF at 0–15 °C, decreasing from about 55% to 35%, and higher at 20–40 °C. This indicated that the NRPO shared similar plasticity with AMF but had higher heat-stable properties. The different SFCs of the NRPO and the HRPO resulted from their TAGs, which contained palmitic acids. MKF exhibited the highest SFCs from 0 to 25 °C, ranging from 61.3% to 20.3%, making it possible to improve the heat-stable properties of the RPOs at room temperature by physical blending [56].

### 3.5. Compatibilities of Binary Blends Consisting of RPOs and MKF

**Figure 2:**
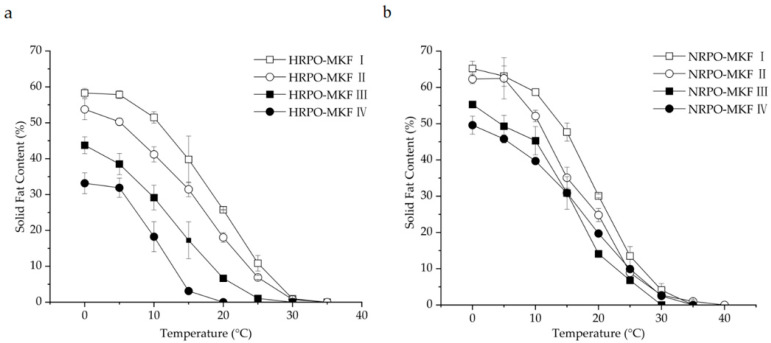
Solid-fat contents of binary blends consisting of MKF with (**a**) HRPO and (**b**) NRPO. HRPO-MKF I, II, III, and IV represent HRPOs accounting for 20%, 40%, 60%, and 80% in the binary blends, respectively. NRPO-MKF I, II, III, and IV represent NRPOs accounting for 20%, 40%, 60%, and 80% in the binary blends, respectively.

**Figure 3:**
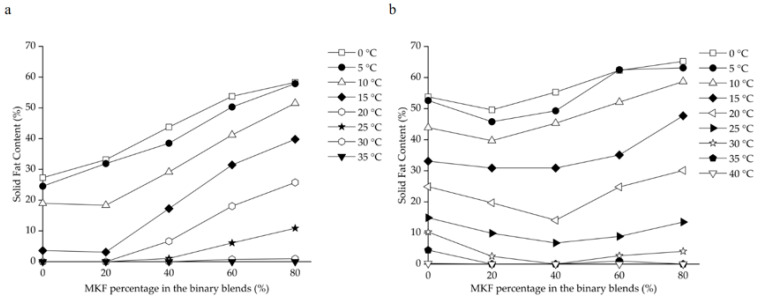
Iso-solid phase diagrams for binary blends consisting of MKF with (**a**) HRPO and (**b**) NRPO.

The SFC changes of binary blends consisting of RPOs and MKF are shown in Figure 2. The heat-stable properties were significantly improved in all the HRPO-MKF blends, and the SFCs decreased rapidly when heated, which is useful in acquiring good mouthfeel [57]. The compatibilities of the binary blends can be studied by determining the SFC changes in the solid percentages with various proportions of the original fat [58]. A binary diagram of the mixtures containing HRPO and MKF showed that the SFC values increased in the concentrations of MKF (Figure 3a). Similar behaviors were observed in the binary blends consisting of hard fat with palm oils, soybean oil, cotton oil, and palm kernel oil [59,60]. The plasticity capabilities showed the degree of fat passing from a solid to a liquid at measured temperatures. The plasticity capabilities of the binary blends at room temperature were also improved, based on different blending ratios. For example, the SFC values of the HRPO without MKF was between around 27% at 0 °C and 0% at 20 °C. The values of the binary blends containing 60–80% MKF gradually decreased from 55–60% at 0 °C to around 10% at 25 °C (Figure 2a), making the blends suitable for the production of heat-stable cream substitutes [61].

The eutectic behaviors of the blends were observed from the curves of NRPO-MKF blends, as shown in Figure 3b. At temperatures above 15 °C, the SFCs of the blends containing 20% and 40% MKF decreased to some extent. This was probably because the melting points of the TAG mixtures were lower than those of their individual components, resulting in lower SFCs at the same temperatures [62]. In this regard, the fat blends, especially NRPO-MKF III, may not be ideal mixtures. In contrast, higher amounts of MKF improved miscibility. The blends containing 20–40% NRPO and 60–80% MKF (i.e., NRPO-MKF I and NRPO-MKF II) exhibited steep SFC profiles (Figure 2b), with the highest SFC obtained at temperatures below 20 °C (24.8–30.1% at 20 °C) and the lowest SFC found at temperatures beyond 25 °C (0.0–1.0% at 35 °C), which exhibited a melt-in-the-mouth property and suggesting that they are the potential specialty fats [44].

### 3.6. Determination of Fractal Dimensions

Fractal dimension (D*f*) can be used as a quantitative indicator to evaluate the spatial distribution of fat crystals [63]. Table 6 shows the fractal dimensions of different fat samples. The high SFC values of MKF indicated that the fat would appear to be solid with a hard texture at temperatures of 15–20 °C, which was helpful in enhancing the ability to crystallize and contributed to a higher fractal dimension [64]. There were no significant differences observed in the D*f* between HRPO (D*f* = 1.008 ± 0.018) and HRPO-MKF II (D*f* = 0.951 ± 0.009). The spatial distribution was further improved by blending 80% HRPO and 20% MKF. In contrast, the addition of MKF in the NRPO-MKF blends affected the formation of small and thin crystals, resulting in decreases in both crystal number and distribution order. 

### 3.7. Whipping Capabilities of Aerated Emulsions

#### 3.7.1. Whipping Time, Overrun, and Serum Loss

Whipping properties reflect the capacities to form a foam system from aerated emulsions. Table 7 illustrates the whipping capabilities of the aerated emulsions formulated with RPOs and MKF. There were no significant differences in the whipping times of the aerated emulsions made by individual HRPOs or MKF. They were still fluid and failed to be shaped after whipping of more than 300 s. This could be explained by the fact that few fat crystals in the HRPO failed to pierce the fat globule membrane to induce the occurrence of partial coalescence. For the emulsions containing both NRPO and MKF, the whipping times were recorded as 180.00–242.50 s, which were less than those of the NRPO-emulsion (299.50 s). It can be concluded that the dense and thin crystals resulted in an increased partial coalescence of the fat globules [65].

Overrun and serum loss are the indices used to evaluate foam quality. Overrun reflects the stabilities and firmness of whipped cream, which is determined by the concentration of serum protein and the degree of partial coalescence [66]. The samples of HRPO and HRPO-MKF IV could not be shaped and had lower overruns (94.90–146.43%) after whipping. In contrast, the overrun of the whipped creams containing NRPO and MKF exhibited higher values, from 228.91% to 233.50%. 

Serum loss is an effective index to evaluate the stability of whipped cream. The whipped cream of NRPO-MKF II had a smooth surface and maintained a firm shape with no serum loss after storage at an ambient temperature for 2 h. In contrast, the samples containing both HRPO and MKF had higher serum losses (14.28–31.15%), which were related to their sparse fat crystals and their weak crystal networks in stabilizing the foam system.

#### 3.7.2. Decorating Performance and Foam Structure

**Figure 4:**
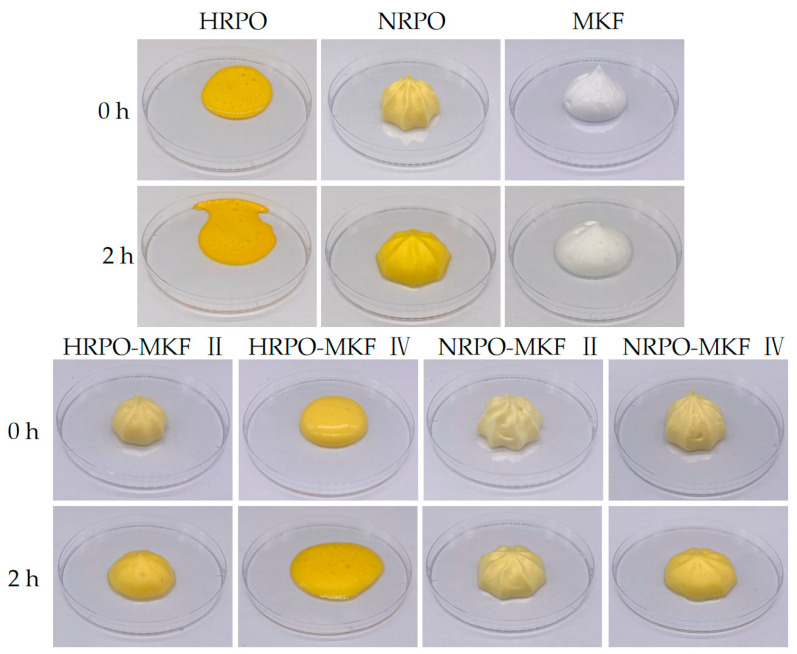
Shaping abilities and appearance of the whipped creams prepared from MKF, RPOs, and their binary blends. HRPO-MKF II represents HRPO accounting for 40% in the binary blends. HRPO-MKF IV represents HRPO accounting for 80% in the binary blends. NRPO-MKF II represents NRPO accounting for 40% in the binary blends. NRPO-MKF IV represents NRPO accounting for 80% in the binary blends.

Decorating performance is shown in Figure 4. There were no obvious foam structures made from 80% HRPO and 20% MKF. The shaping abilities of the whipped samples with HRPO-MKF II (containing 60% MKF) and NRPO were significantly improved and the texture became clearer. This indicated that the addition of MKF could improve shaping abilities significantly. This is closely related to the dense fat crystal structures of MKF. In addition, the blends of NRPO and MKF contributed to improving the shaping abilities of the aerated emulsions.

**Figure 5:**
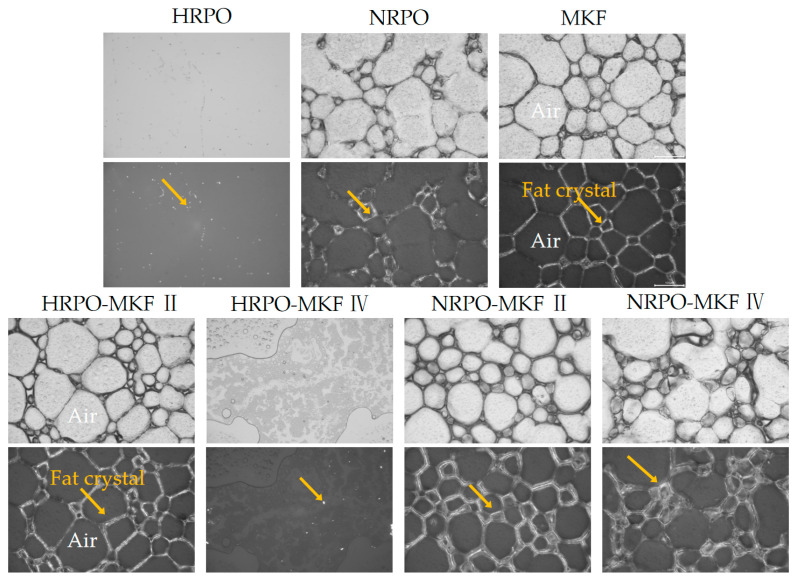
Polarized light micrographs (**bottom**) and the corresponding optical micrographs (**top**) for the whipped creams (the scale bar indicates 100 μm) of whipped creams. The yellow arrows point to fat crystals. HRPO-MKF II represents HRPO accounting for 40% in the binary blends. HRPO-MKF IV represents HRPO accounting for 80% in the binary blends. NRPO-MKF II represents NRPO accounting for 40% in the binary blends, NRPO-MKF IV represents NRPO accounting for 80% in the binary blends.

The sizes, distributions, and morphologies of fat crystals were crucial in determining the rigidity and stability of the foam [67]. Figure 5 reveals crystal networks of the foam structures after whipping. Except for the samples of HRPO and HRPO-MKF IV, the emulsions formed clear three-dimensional crystal networks. The higher strengths in network structures were observed in samples of MKF and NRPO-MKF II, which were consistent in their dense and thin crystals. The MKF contained 36.78–41.40% of stearic acid, and some large fat crystals that existed in the network assisted the formation of a stronger network, which could stabilize the foams against coalescence during storage [60,63].

### 3.8. Texture Profile Analysis

Texture properties are crucial for the processing properties and the eating quality of food products. Firmness is the maximum force that occurs during the first compression, which is an effective index to evaluate the stability of whipped cream. As shown in Table 8, the firmness of the sample formulated by MKF was 32.41 g, and the index values were decreased by addition of HRPO (14.49–18.63 g) and increased with the effects of NRPO (36.91–40.04 g). Consistency has a great effect on the eating quality and acceptability of the final product [68]. The whipped cream prepared from MKF had a higher consistency (963.72 g·s). The samples formulated with 40–100% of NRPO resulted in an increase in consistency values (779.34–1269.23 g·s) compared with those of the samples substituted by 40–100% HRPO (256.60–431.12 g·s). Cohesiveness represents the extent to which a material can be deformed before it ruptures [69]. The cohesiveness of the MKF sample was 16.80 g, which was significantly higher than those of the HRPO-MKF samples (9.58–10.17 g). However, when 40–80% NRPO was added to the MKF sample, the cohesiveness increased to 20.23–25.67 g. It was found that all the textural characteristic values were improved in the samples incorporating NRPO with MKF. This could be explained by the fact that the viscous liquid became a plastic foam structure during whipping, which depended to a large extent on the partial coalescence of the fat globules [70].

The correlation analysis of fatty acid characteristics and indicators of the samples is shown in Table 9. Overrun showed significant positive correlations with S/O (the ratio of steric acid and oleic acid content; r = 0.597) and LCFA content (long carbon chain-fatty acid; r = 0.650). Serum loss and cohesiveness showed significant negative correlations with overrun. Furthermore, overrun had highly significant positive correlations with texture-related indices, mainly firmness and consistency. This indicated that the regulation of S/O and LCFA contributed to improving overrun and firmness, as well as decreasing serum loss [71]. 

## 4. Conclusions

RPOs are one of the most promising food ingredients, owing to their unique triacylglycerol compositions and their high amounts of micronutrients, especially squalene and total polyphenol, which contribute to their desirable OSIs. Their oxidative stabilities were found to be significantly higher than those of most of the common edible oils. In this regard, RPOs could meet requirements for healthy ingredients in daily diets. In particular, HRPO could be used as an edible oil in cooking food, due to its low melting behaviors. Although their heat-stable properties at room temperature may limit the usages of RPOs, such properties could be improved by incorporating RPOs with MKF. The binary blends with suitable ratios of RPOs to MKF exhibited desirable compatibilities, making the blends suitable for the manufacture of aerated emulsions. In particular, the whipping performance and the foam-heat stabilities of the NRPO emulsions were significantly improved by incorporating NRPO with MKF. The results provide for new applications of RPOs and MKF in aerated emulsions, with desired whipping capabilities.

## Figures and Tables

**Table 1:** Fatty-acid compositions of red palm oils, mango kernel fat, and their binary blends.

Fatty Acids (%)	HRPO	NRPO	MKF	HRPO-MKF II	HRPO-MKF IV	NRPO-MKF II	NRPO-MKF IV
C12:0	4.36 ± 0.05 ^a^	0.22 ± 0.01 ^d^	N.D.	1.78 ± 0.01 ^c^	3.44 ± 0.04 ^b^	N.D.	N.D.
C14:0	2.33 ± 0.02 ^a^	0.90 ± 0.02 ^d^	N.D.	0.99 ± 0.01 ^c^	1.88 ± 0.01 ^b^	0.39 ± 0.02 ^f^	0.73 ± 0.01 ^e^
C16:0	34.95 ± 0.34 ^c^	42.96 ± 0.27 ^a^	6.28 ± 0.10 ^g^	18.59 ± 0.00 ^f^	29.88 ± 0.04 ^d^	21.33 ± 0.03 ^e^	35.78 ± 0.01 ^b^
C18:0	3.87 ± 0.01 ^g^	5.83 ± 0.01 ^f^	47.19 ± 0.31 ^a^	28.44 ± 0.12 ^c^	11.96 ± 0.03 ^e^	30.31 ± 0.17 ^b^	14.00 ± 0.06 ^d^
C18:1	42.27 ± 0.41 ^a^	38.99 ± 0.25 ^e^	38.16 ± 0.19 ^f^	40.60 ± 0.06 ^c^	41.76 ± 0.03 ^b^	38.58 ± 0.13 ^d,e^	38.98 ± 0.04 ^d^
C18:2	11.57 ± 0.11 ^a^	10.70 ± 0.07 ^b^	2.59 ± 0.04 ^g^	6.42 ± 0.00 ^e^	9.79 ± 0.01 ^c^	5.99 ± 0.04 ^f^	9.16 ± 0.01 ^d^
C18:3	0.55 ± 0.03 ^a^	N.D.	N.D.	N.D.	N.D.	N.D.	N.D.
C20:0	N.D.	N.D.	5.78 ± 0.03 ^a^	3.21 ± 0.04 ^c^	1.30 ± 0.04 ^d^	3.41 ± 0.14 ^b^	1.36 ± 0.00 ^d^
∑SFA	46.05 ± 0.42 ^e^	49.91 ± 0.32 ^c,d,e^	56.37 ± 4.31 ^a^	52.99 ± 0.07 ^a,b,c^	48.45 ± 0.02 ^d,e^	55.05 ± 0.06 ^a,b^	51.86 ± 0.05 ^b,c,d^
∑MUFA	42.27 ± 0.41 ^a^	38.99 ± 0.25 ^d^	38.16 ± 0.19 ^e^	40.60 ± 0.06 ^b^	41.76 ± 0.03 ^a^	38.58 ± 0.13 ^c^	38.98 ± 0.04 ^c,d^
∑PUFA	12.13 ± 0.13 ^a^	10.70 ± 0.07 ^b^	2.59 ± 0.04 ^g^	6.42 ± 0.00 ^e^	9.79 ± 0.01 ^c^	5.99 ± 0.04 ^f^	9.16 ± 0.01 ^d^

HRPO, H. red palm oil; NRPO, N. red palm oil; MKF, mango kernel fat; HRPO-MKF II and IV represent HRPO accounting for 40% and 80% in the binary blends, respectively. NRPO-MKF II and IV represent NRPO accounting for 40% and 80% in the binary blends respectively. SFA, saturated fatty acid; MUFA, monounsaturated fatty acid; PUFA, polyunsaturated fatty acid. Values followed by different letters in the same row represent significant differences at *p* < 0.05.

**Table 2:** Triacylglycerol compositions of red palm oils, mango kernel fat, and their binary blends.

TAGs (%)	HRPO	NRPO	MKF	HRPO-MKF II	HRPO-MKF IV	NRPO-MKF II	NRPO-MKF IV
LaLaLa	5.18 ± 0.69 ^a^	1.65 ± 0.17 ^b^	N.D.	1.93 ± 0.16 ^b^	4.09 ± 0.81 ^a^	1.17 ± 0.09 ^b^	1.58 ± 0.56 ^b^
LaLaM	8.36 ± 1.12 ^a^	4.43 ± 0.55 ^c^	N.D.	3.34 ± 0.45 ^c^	6.65 ± 0.18 ^b^	1.50 ± 0.66 ^d^	4.05 ± 0.42 ^c^
LaMM	3.42 ± 0.37 ^d^	1.77 ± 0.22 ^f^	7.30 ± 0.19 ^a^	5.32 ± 0.10 ^b^	4.68 ± 0.16 ^c^	4.77 ± 0.36 ^b,c^	2.73 ± 0.08 ^e^
PPP	4.57 ± 0.35 ^c^	10.83 ± 1.30 ^a^	N.D.	1.80 ± 0.10 ^d^	3.62 ± 0.40 ^c^	4.65 ± 0.26 ^c^	8.71 ± 0.16 ^b^
POP	22.42 ± 0.38 ^c^	29.31 ± 1.05 ^a^	2.08 ± 0.13 ^g^	10.20 ± 0.21 ^f^	18.50 ± 0.37 ^d^	13.18 ± 0.11 ^e^	24.02 ± 0.37 ^b^
PLP	15.15 ± 1.00 ^a^	15.12 ± 1.44 ^a^	N.D.	6.78 ± 0.28 ^c^	12.56 ± 0.30 ^b^	6.22 ± 0.15 ^c^	12.00 ± 0.35 ^b^
POS	1.91 ± 0.21 ^e^	2.47 ± 0.18 ^d,e^	7.64 ± 0.47 ^a^	5.23 ± 0.37 ^b^	3.05 ± 0.01 ^d^	5.79 ± 0.23 ^b^	3.91 ± 0.42 ^c^
POO	23.10 ± 0.43 ^a^	18.39 ± 1.20 ^b^	6.54 ± 0.64 ^f^	12.72 ± 0.54 ^d^	19.36 ± 0.25 ^b^	11.21 ± 0.17 ^e^	15.63 ± 0.45 ^c^
POL	10.11 ± 1.98 ^a^	7.92 ± 0.59 ^b^	N.D.	3.99 ± 0.21 ^c^	8.33 ± 0.60 ^a,b^	3.35 ± 0.58 ^c^	6.41 ± 0.16 ^b^
SOS	1.82 ± 0.18 ^d^	2.06 ± 0.32 ^d^	31.91 ± 0.83 ^a^	19.43 ± 0.54 ^b^	7.88 ± 0.54 ^c^	19.83 ± 0.14 ^b^	7.48 ± 0.38 ^c^
SOO	3.33 ± 0.28 ^d^	3.48 ± 0.33 ^d^	23.86 ± 1.07 ^a^	15.12 ± 0.41 ^b^	7.29 ± 0.44 ^c^	15.09 ± 0.06 ^b^	7.26 ± 0.28 ^c^
OOO	0.68 ± 0.12 ^e^	3.08 ± 0.64 ^c,d^	9.19 ± 1.55 ^a^	6.95 ± 0.12 ^b^	2.47 ± 0.27 ^d^	7.72 ± 0.14 ^a,b^	4.41 ± 0.46 ^c^
SLO	N.D.	N.D.	4.03 ± 0.39 ^a^	2.54 ± 0.27 ^b^	0.67 ± 0.23 ^c^	2.02 ± 0.84 ^b^	0.55 ± 0.01 ^c^
OLO	N.D.	N.D.	5.00 ± 0.06 ^a^	3.04 ± 0.23 ^b^	0.90 ± 0.04 ^d^	2.61 ± 0.40 ^b^	0.91 ± 0.10 ^d^
OLL	N.D.	N.D.	2.46 ± 0.72 ^a^	1.64 ± 0.27 ^b^	N.D.	0.97 ± 0.13 ^b,c^	0.49 ± 0.08 ^c,d^

TAGs, triacylglycerols; HRPO, H. red palm oil; NRPO, N. red palm oil; MKF, mango kernel fat; HRPO-MKF II and IV represent HRPO accounting for 40% and 80% in the binary blends, respectively. NRPO-MKF II and IV represent NRPO accounting for 40% and 80% in the binary blends, respectively. Fatty acid in TAG: Ca, 10:0; La, 12:0; M, 14:0; P, 16:0; S, 18:0; O, 18:1 9c; L, 18:2 9c,12c; A, 20:0. Values followed by different letters in the same row represent significant differences at *p* < 0.05.

**Table 3:** Sterol, squalene, tocopherol, carotene, and total polyphenol of red palm oils.

Micronutrients (mg/kg)	HRPO	NRPO
Sterol		
Campesterol	142.03 ± 17.91 ^b^	116.61 ± 7.47 ^b^
Stigmasterol	74.29 ± 85.88 ^b^	75.48 ± 6.88 ^c^
Sitosterol	440.85 ± 34.32 ^a^	196.06 ± 1.02 ^a^
Total	662.96 ± 60.42	388.14 ± 1.60
Squalene	28.05 ± 2.42	52.80 ± 1.69
Tocopherol		
α-Tocopherol	316.70 ± 4.17 ^a^	137.23 ± 10.85 ^a^
β-Tocopherol	22.85 ± 3.18 ^c^	28.28 ± 2.86 ^b^
γ-Tocopherol	117.00 ± 1.13 ^b^	132.00 ± 10.61 ^a^
δ-Tocopherol	16.88 ± 5.55 ^c^	30.43 ± 1.31 ^b^
Total	473.43 ± 0.67	327.93 ± 17.29
Carotene	154.04 ± 2.77	91.48 ± 0.96
Total polyphenol	45.00 ± 1.02	50.00 ± 1.77

Values followed by different letters in the same column represent significant differences at *p* < 0.05.

**Table 4:** Oxidative stability indices of HRPO, NRPO, and their counterparts without micronutrients.

Samples	Time (h)
HRPO	10.02 ± 0.06 ^b^
HRPO counterpart	1.12 ± 0.17 ^c^
NRPO	12.06 ± 0.27 ^a^
NRPO counterpart	1.82 ± 0.46 ^c^

Values followed by different letters in the same column represent significant differences at *p* < 0.05.

**Table 5:** Pearson correlation analysis for micronutrient levels and oxidative stability indices of RPOs and their counterparts.

Micronutrients	Correlation Coefficient Value (r)				
	Sterol	Squalene	Tocopherol	Carotene	Total Polyphenol
OSI	−0.963 *	0.991 **	−0.969 *	−0.989 *	0.977 *
OSI (counterpart)	−0.797	0.837	−0.699	−0.760	0.915

* and **, significance and high significance at *p* < 0.05 and *p* < 0.01, respectively.

**Table 6:** Fractal dimensions of RPOs, MKF, and their binary blends.

Samples	Fractal Dimension
HRPO	1.008 ± 0.018 ^c,d^
NRPO	1.206 ± 0.030 ^b^
MKF	1.220 ± 0.044 ^b^
HRPO-MKF II	0.951 ± 0.009 ^d^
HRPO-MKF IV	1.337 ± 0.028 ^a^
NRPO-MKF II	1.024 ± 0.023 ^c,d^
NRPO-MKF IV	1.029 ± 0.033 ^c^

HRPO-MKF II represents HRPO accounting for 40% in the binary blends. HRPO-MKF IV represents HRPO accounting for 80% in the binary blends. NRPO-MKF II represents NRPO accounting for 40% in the binary blends. NRPO-MKF IV represents NRPO accounting for 80% in the binary blends. Values followed by different letters in the same column represent significant differences at *p* < 0.05.

**Table 7:** Whipping properties of the aerated emulsions formulated with RPOs, MKF, and their binary blends.

Samples	Whipping Time (s)	Overrun (%)	Serum Loss (%)
HRPO	363.00 ± 4.24 ^a^	94.90 ± 5.97 ^d^	34.12 ± 5.06 ^a^
NRPO	299.50 ± 0.71 ^b^	216.90 ± 0.00 ^a,b^	8.67 ± 0.21 ^c^
MKF	360.5 ± 0.71 ^a^	260.25 ± 8.84 ^a,b^	6.96 ± 0.33 ^c^
HRPO-MKF II	361.50 ± 0.71 ^a^	183.42 ± 35.07 ^b,c^	14.28 ± 2.43 ^b^
HRPO-MKF IV	362.00 ± 1.41 ^a^	146.43 ± 30.30 ^c^	31.15 ± 1.25 ^a^
NRPO-MKF II	242.50 ± 3.54 ^c^	233.50 ± 10.48 ^a^	0.00 ± 0.00 ^d^
NRPO-MKF IV	180.00 ± 5.66 ^d^	228.91 ± 16.98 ^a,b^	3.61 ± 0.86 ^c,d^

HRPO-MKF II represents HRPO accounting for 40% in the binary blends. HRPO-MKF IV represents HRPO accounting for 80% in the binary blends. NRPO-MKF II represents NRPO accounting for 40% in the binary blends, NRPO-MKF IV represents NRPO accounting for 80% in the binary blends. Values followed by different letters in the same column represent significant differences at *p* < 0.05.

**Table 8:** Textural characteristic of the whipped creams formulated from MKF, RPOs, and their binary blends.

Samples	Firmness (g)	Consistency (g·s)	Cohesiveness (g)
HRPO	17.15 ± 1.77 ^b^	360.18 ± 18.14 ^d^	5.56 ± 0.35 ^c^
NRPO	31.17 ± 3.25 ^a^	779.34 ± 87.42 ^b,c^	17.09 ± 1.60 ^a,b,c^
MKF	32.41 ± 3.55 ^a^	963.72 ± 209.43 ^a,b^	16.80 ± 4.38 ^a,b,c^
HRPO-MKF II	18.63 ± 3.13 ^b^	431.12 ± 70.7 ^c,d^	10.17 ± 2.37 ^b,c^
HRPO-MKF IV	14.49 ± 0.41 ^b^	256.60 ± 33.80 ^d^	9.58 ± 3.08 ^b,c^
NRPO-MKF II	40.04 ± 1.36 ^a^	1269.23 ± 72.14 ^a^	20.23 ± 1.89 ^a,b^
NRPO-MKF IV	36.91 ± 5.09 ^a^	1087.40 ± 138.33 ^a,b^	25.67 ± 7.33 ^a^

HRPO-MKF II represents HRPO accounting for 40% in the binary blends. HRPO-MKF IV represents HRPO accounting for 80% in the binary blends. NRPO-MKF II represents NRPO accounting for 40% in the binary blends. NRPO-MKF IV represents NRPO accounting for 80% in the binary blends. Values followed by different letters in the same column represent significant differences at *p* < 0.05.

**Table 9:** The correlation analysis of fatty acid characteristics and indicators of aerated emulsion prepared by fat samples.

	L/O	P/O	S/O	MCFA	LCFA	UFA	Overrun	Serum Loss	Firmness	Consistency	Cohesiveness
L/O	1.00										
P/O	0.185	1.00									
S/O	−0.737 **	−0.774 **	1.00								
MCFA	0.972 **	0.325	−0.802 *	1.00							
LCFA	−0.920 **	−0.532 *	0.921 **	−0.945 **	1.00						
UFA	0.805 **	0.664 **	−0.960 **	0.869 **	−0.952 **	1.00					
Overrun	−0.649 *	−0.338	0.597 *	−0.631 *	0.650 *	−0.544 *	1.00				
Serum loss	0.458	0.148	−0.339	0.425	−0.440	0.330	−0.868 **	1.00			
Firmness	−0.517	−0.062	0.297	−0.496	0.459	−0.336	0.862 **	−0.871 **	1.00		
Consistency	−0.517	−0.044	0.297	−0.491	0.459	−0.336	0.862 **	−0.904 **	0.991 **	1.00	
Cohesiveness	0.449	0.251	−0.383	0.422	−0.475	0.304	−0.858 **	0.790 **	−0.858 **	−0.847 **	1.00

L/O, the ratio of lauric acid and oleic acid content; P/O, the ratio of palmitic acid and oleic acid content; S/O, the ratio of steric acid and oleic acid content; MCFA: medium carbon chain-saturated fatty acids; LCFA, long carbon chain-saturated fatty acids; UFA, unsaturated fatty acids; * and **, significance and high significance at *p* < 0.05 and *p* < 0.01, respectively.

## Data Availability

Data is contained within the article.
